# Author Correction: Genome-wide association study of knee pain identifies associations with *GDF5* and *COL27A1* in UK Biobank

**DOI:** 10.1038/s42003-020-0880-x

**Published:** 2020-03-26

**Authors:** Weihua Meng, Mark J. Adams, Colin N. A. Palmer, The 23andMe Research Team, Michelle Agee, Babak Alipanahi, Robert K. Bell, Katarzyna Bryc, Sarah L. Elson, Pierre Fontanillas, Nicholas A. Furlotte, Barry Hicks, David A. Hinds, Karen E. Huber, Ethan M. Jewett, Yunxuan Jiang, Aaron Kleinman, Keng-Han Lin, Nadia K. Litterman, Jennifer C. McCreight, Matthew H. McIntyre, Kimberly F. McManus, Joanna L. Mountain, Elizabeth S. Noblin, Carrie A. M. Northover, Steven J. Pitts, G. David Poznik, J. Fah Sathirapongsasuti, Janie F. Shelton, Suyash Shringarpure, Chao Tian, Joyce Y. Tung, Vladimir Vacic, Xin Wang, Catherine H. Wilson, Jingchunzi Shi, Adam Auton, Kathleen A. Ryan, Joanne M. Jordan, Braxton D. Mitchell, Rebecca D. Jackson, Michelle S. Yau, Andrew M. McIntosh, Blair H. Smith

**Affiliations:** 1 Division of Population Health and Genomics, School of Medicine, University of Dundee, Dundee, UK; 2 Division of Psychiatry, Edinburgh Medical School, University of Edinburgh, Edinburgh, UK; 3 23andMe, Inc., Mountain View, CA USA; 4 Department of Medicine, University of Maryland School of Medicine, Baltimore, MD USA; 5 Department of Medicine, University of North Carolina School of Medicine, Chapel Hill, NC USA; 6 Geriatric Research, Education and Clinical Center, Veterans Affairs Medical Center, Baltimore, MD USA; 7 Division of Endocrinology, Diabetes and Metabolism, The Ohio State University, Columbus, OH USA; 8 Institute for Aging Research, Hebrew SeniorLife, Harvard Medical School, Boston, MA USA

**Keywords:** Pain, Musculoskeletal system, Genome-wide association studies

Correction to: *Communications Biology* 10.1038/s42003-019-0568-2, published online 28 August 2019.

In the original published version of this article, the alleles shown in the Table 2 column labelled ‘Effective allele in all cohorts’ was incorrect due to SNP concordance issues. This error has now been corrected in the HTML and PDF versions.

