# Supplementary material for: Genome-wide association study of knee pain identifies associations with GDF5 and COL27A1 in UK Biobank
Source: Commun Biol. 2019 Aug 28;2:321. doi: 10.1038/s42003-019-0568-2 (PMC6713725; doi:10.1038/s42003-019-0568-2)
Supplement: Supplementary file 1 — Supplementary Information [file 42003_2019_568_MOESM1_ESM.pdf]

Supplementary Figures

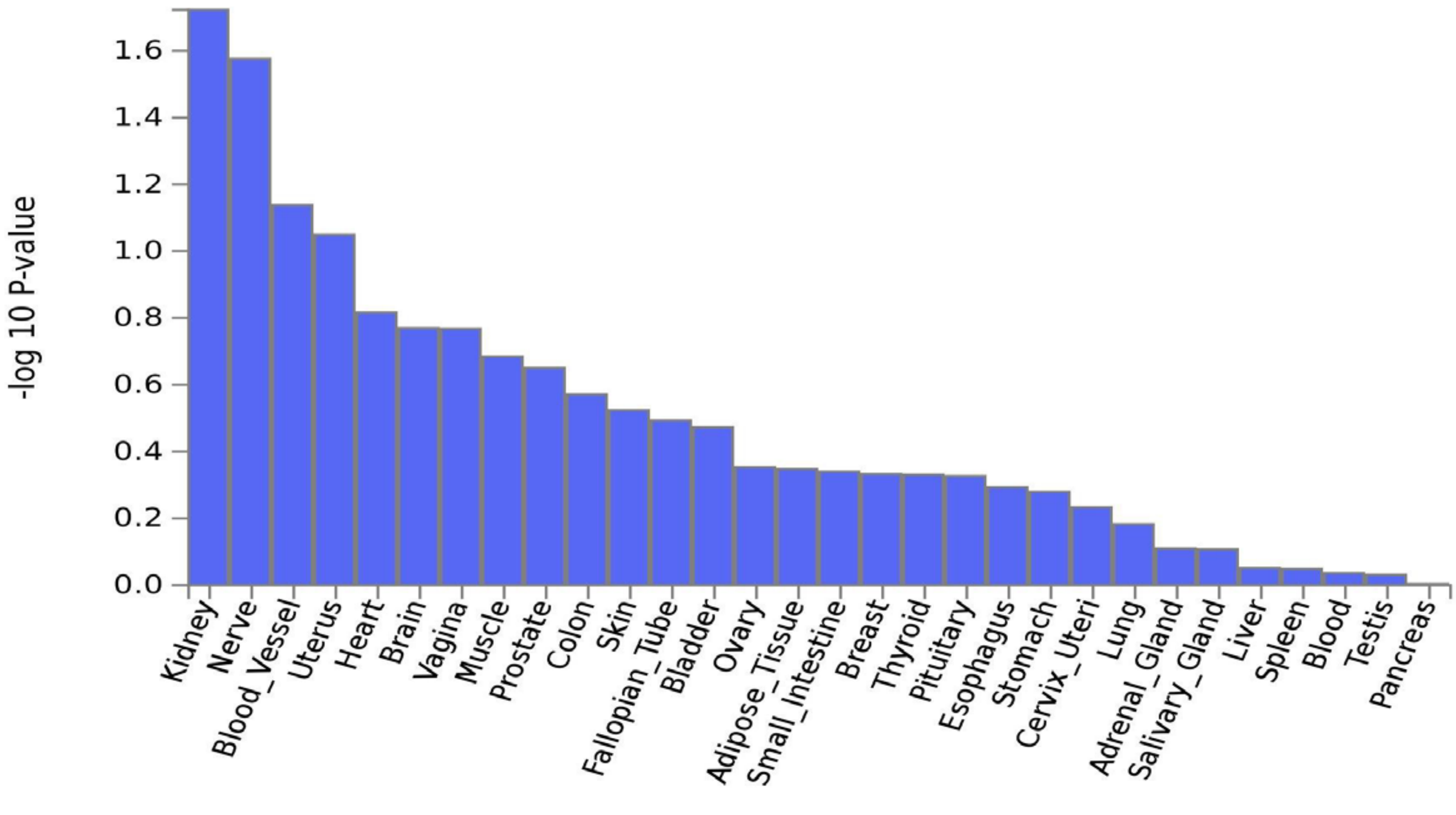

Supplementary figure 1 the tissue expression analysis of 30 general tissues by FUMA

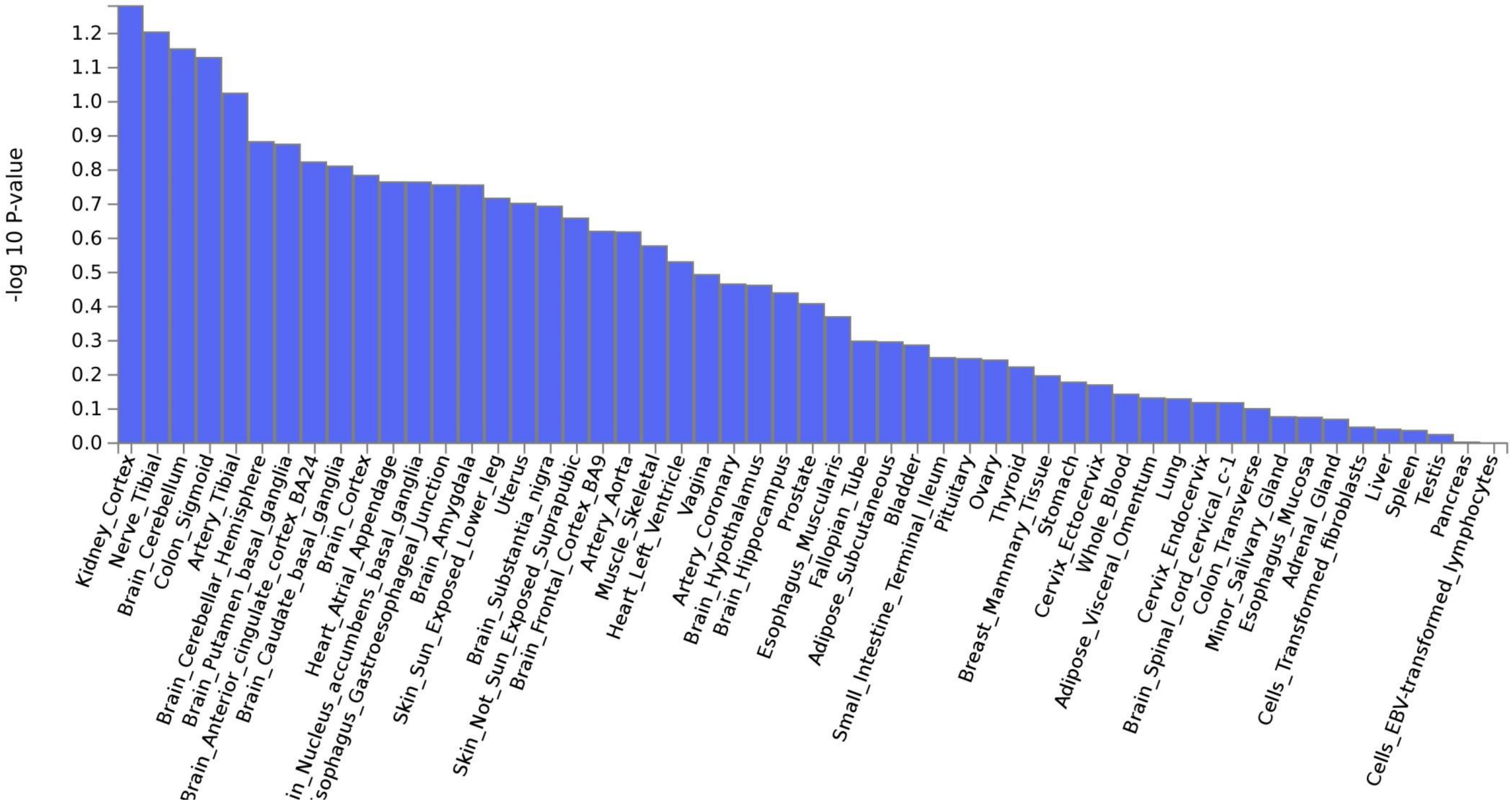

Supplementary figure 2 the tissue expression analysis of 53 specific tissues by FUMA

Supplementary Tables

Supplementary Table 1: the 11 associated genes of the gene analysis by FUMA

Gene	Chromosome	Start	Stop	Number of SNPs	Z-STAT	*P*
GDF5	20	34021145	34042568	66	6.6939	1.09E-11
UQCC1	20	33890369	33999944	358	6.6671	1.30E-11
CEP250	20	34042985	34099804	189	6.3487	1.09E-10
PODXL	7	131185021	131242976	297	5.2024	9.84E-08
C20orf173	20	34111014	34117481	18	5.1655	1.20E-07
SPAG4	20	34203814	34208971	15	5.0917	1.77E-07
MTMR3	22	30279144	30426855	623	4.8555	6.00E-07
ERGIC3	20	34129770	34145405	50	4.8223	7.10E-07
FBLN2	3	13573824	13679922	701	4.6333	1.80E-06
CPNE1	20	34213953	34252878	143	4.5625	2.53E-06
CDC42SE2	5	130581186	130734140	508	4.5372	2.85E-06

Supplementary Table 2: the top 10 gene sets generated by FUMA

Full name of the pathways	Number of genes	Beta	Beta-STD	SE	*P*
Curated_gene_sets:nikolsky_breast_cancer_20q11_amplico	31	1.44	0.0605	0.268	$3.76x10^{-8}$
GO_cc:go_presynaptic_active_zone	25	0.765	0.0288	0.187	$2.16x10^{-5}$
Curated_gene_sets:biocarta_tid_pathway	13	0.776	0.0211	0.222	0.000237
GO_cc:go_anchored_component_of_external_side_of_plasma_membrane	19	0.697	0.0229	0.202	0.000278
Curated_gene_sets:figueroa_aml_methylation_cluster_7_dn	5	1.48	0.025	0.433	0.000317
GO_cc:go_intrinsic_component_of_external_side_of_plasma_membrane	23	0.615	0.0222	0.183	0.000402
Curated_gene_sets:traynor_rett_syndrom_dn	15	0.746	0.0218	0.229	0.000548
Curated_gene_sets:kegg_tgf_beta_signaling_pathway	75	0.32	0.0208	0.0986	0.000598
GO_bp:go_embryonic_camera_type_eye_development	32	0.537	0.0229	0.17	0.000797
GO_bp:go_mating_behavior	22	0.589	0.0208	0.188	0.000877

STD: Standard deviation

SE: Standard error

Supplementary Table 3. Distribution by sex and current age for individuals included in the 23andMe full GWAS

Phenotype	Group	Total	Male	Female	(0,30]	(30,45]	(45,60]	(60,Inf]
osteoarthritis	case	253880	80576	173304	2999	17468	61923	171490
	control	1286245	605442	680803	219308	371034	351348	344555

Supplementary Table 4. Distribution across 23andMe genotyping platforms for individuals included in the full GWAS

Group	Total	v1/v2	v3	v4	v5
osteoarthritis case	253880	2464	21512	116307	113597
osteoarthritis control	1286245	9873	83618	535550	657204
