# Supplementary material for: Genome-wide association study of knee pain identifies associations with GDF5 and COL27A1 in UK Biobank
Source: Commun Biol. 2019 Aug 28;2:321. doi: 10.1038/s42003-019-0568-2 (PMC6713725; doi:10.1038/s42003-019-0568-2)
Supplement: Supplementary file 2 — Description of additional supplementary items [file 42003_2019_568_MOESM2_ESM.docx]

Supplementary Dataset 1

Significantly associated SNPs (N=107) in the discovery stage using the UK Biobank cohort

Supplementary Dataset 2

The genetic correlations between knee pain and other available traits in the LD hub
